# Synthesis of 2-tetrafluoropyridyl-4,5-disubstituted 1,2,3-triazoles

**DOI:** 10.1186/s40064-016-3398-4

**Published:** 2016-11-11

**Authors:** Khalil Beyki, Malek Taher Maghsoodlou, Reza Heydari

**Affiliations:** Department of Chemistry, Faculty of Science, University of Sistan and Baluchestan, P.O. Box 98135-674, Zahedan, Iran

**Keywords:** Pentafluoropyridine, Chalcone, 1,2,3-Triazoles, CuO, ^19^F-NMR

## Abstract

**Abstract:**

By cycloaddition reaction of sodium azide with chalcone in the presence of CuO as a catalyst in DMF a 1,2,3-triazole are prepared in reaction with pentafluoropyridine to give 2-(tetrafluoropyridin-4-yl)-1,2,3-triazole derivatives in good yields and high regioselectivity. The regioselectivity of the compounds are confirmed by ^19^F-NMR and other spectroscopy.

**Graphical Abstract:**

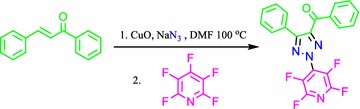

## Background

Recently, perfluorocarbons have been used as building blocks in the pharmaceutical industry and in material science due to their unique properties (Kenneth 2006; Bruce 2001). In pharmacology and medicinal researches, it is common to substitute hydrogen with fluorine atom for increasing the physiochemical (e.g. solubility, stability) and biological activity (e.g. absorption, distribution, metabolism, elimination and toxicity) of drugs (Iwao 2009).

Pentafluoropyridine in which all the hydrogen atoms in pyridine ring have been replaced by fluorine atoms are highly susceptible towards nucleophilic attack owing to the presence of several highly electronegative fluorine atoms and nitrogen hetero atom; consequently, the chemistry of pentafluoropyridine is dominated by nucleophilic aromatic substitution processes and new chemistry continues to emerge (Iwao 2009; Reza et al. 2008; Mark et al. 2013; Van Ba and Donald 2012). The order of nucleophilic attack for pentafluoropyridine is established to be para > ortho > meta positions, so the reactions of pentafluoropyridine with some nucleophile occur selectively at the para position as this site is most activated towards nucleophilic additions to afford of 4-substited tetrafluoropyridine (Hadjar et al. 2001; Matthew et al. 2010; Jingjing et al. 2014).

**Fig. 1:**
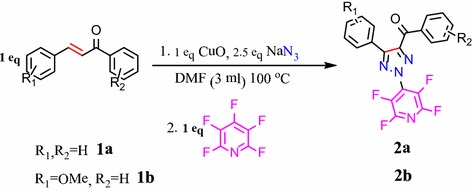
Synthesis of 2-tetrafluoropyridyl-1,2,3-triazoles

Baohua Chen and coworker’s reported the synthesis of *N*-2-aryl-substituted-1,2,3-triazoles and arylation in the last step (Yuanqing et al. 2012). With this goal, we have employed the highly electron-deficient pentafluoropyridine for arylation of 1,2,3-triazoles in the last step for preparation of 2-tetrafluoropyridyl-1,2,3 triazoles (Fig. 1).

Earlier, we reported the synthesis of 4-substituted-2,3,5,6-tetrafluoropyridine derivatives by the reaction of pentafluoropyridine with malononitrile, 1-tetrazole-5-thiol, piperazine (Khalil et al. 2015), hydroxylated naphtoquinones and hydroxylated antraquinones (Khalil et al. 2016).

## Results and discussion

In this research, we describe the synthesis of 2-tetrafluoropyridyl-4,5-disubstituted-1,2,3-triazoles via Diels–Alder cycloaddition reaction of sodium azide and 1,3-diphenylprop-2-en-1-one (chalcone) and then nucleophilic substitution reactions with pentafluoropyridine in DMF as solvent.

**Fig. 2:**
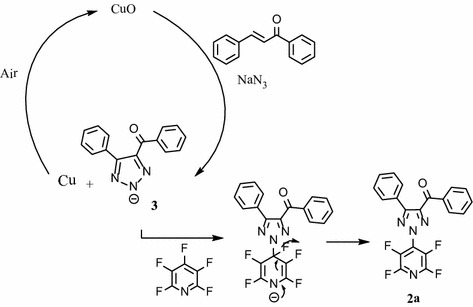
The mechanism for the formation of 2-tetrafluoropyridyl-4,5-disubstituted 1,2,3-triazoles

Reaction of 3-(phenyl)-1-phenylprop-2-en-1-one (chalcone) (**1a**) with sodium azide and one equiv catalyst of CuO in the solvent of DMF gave intermediate of 4-benzoyl-5-phenyl-1,2,3-triazol-2-ide **3** (TLC monitoring during the course of the reaction; Fig. 2). In last step, nitrogen nucleophile of 1,2,3-triazoles (intermediate 3) attack at the most activated 4-position of pentafluoropyridine and elimination of 4-fluor pyridine ring leads to the formation of (2-(perfluoropyridin-4-yl)-5-phenyl-*2H*-1,2,3-triazol-4-yl)(phenyl)methanone **2a** in good yield. In the mechanism of this transformation CuO acted as the oxidant and then Cu was oxidized to CuO by air. In this search, we did not perform any optimization for this protocol and utilize the same condition reported in previous paper (Yuanqing et al. 2012).

Purification of **2a** was achieved by column chromatography using ethyl acetate/*n*-hexane (2:10). The melting point, ^19^F, ^1^H, ^13^C NMR and mass spectra of the obtained product clearly indicated the formation of (2-(perfluoropyridin-4-yl)-5-phenyl-*2H*-1,2,3-triazol-4-yl)(phenyl)methanone **2a**. For example, in the ^1^H NMR spectrum of compound **2a**, the aromatic proton resonances were observed as multiplets at δ = 7.60–8.61 ppm. The ^13^C NMR spectrum of compound **2a** showed 20 distinct resonances consistent with the recommended structure.

**Fig. 3:**
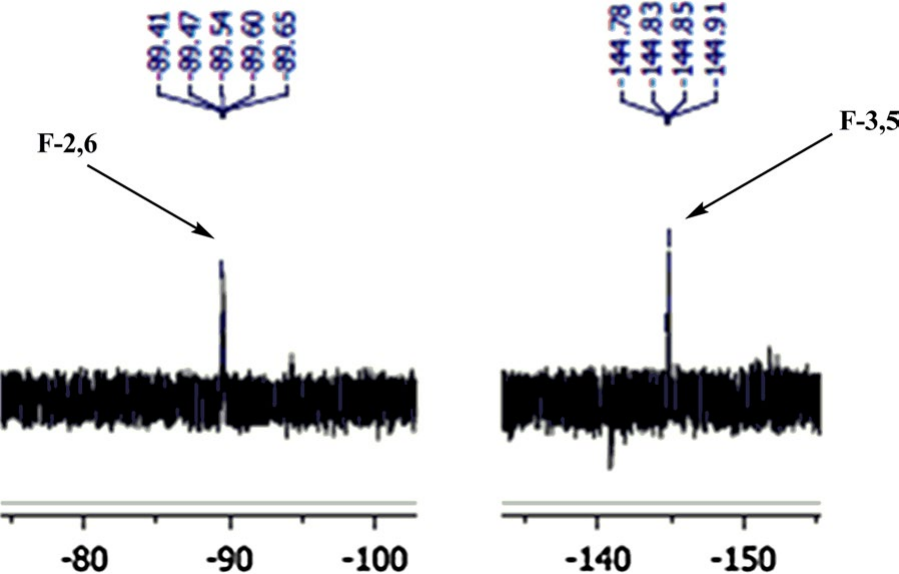
^19^F NMR spectrum of **2a**

**Fig. 4:**
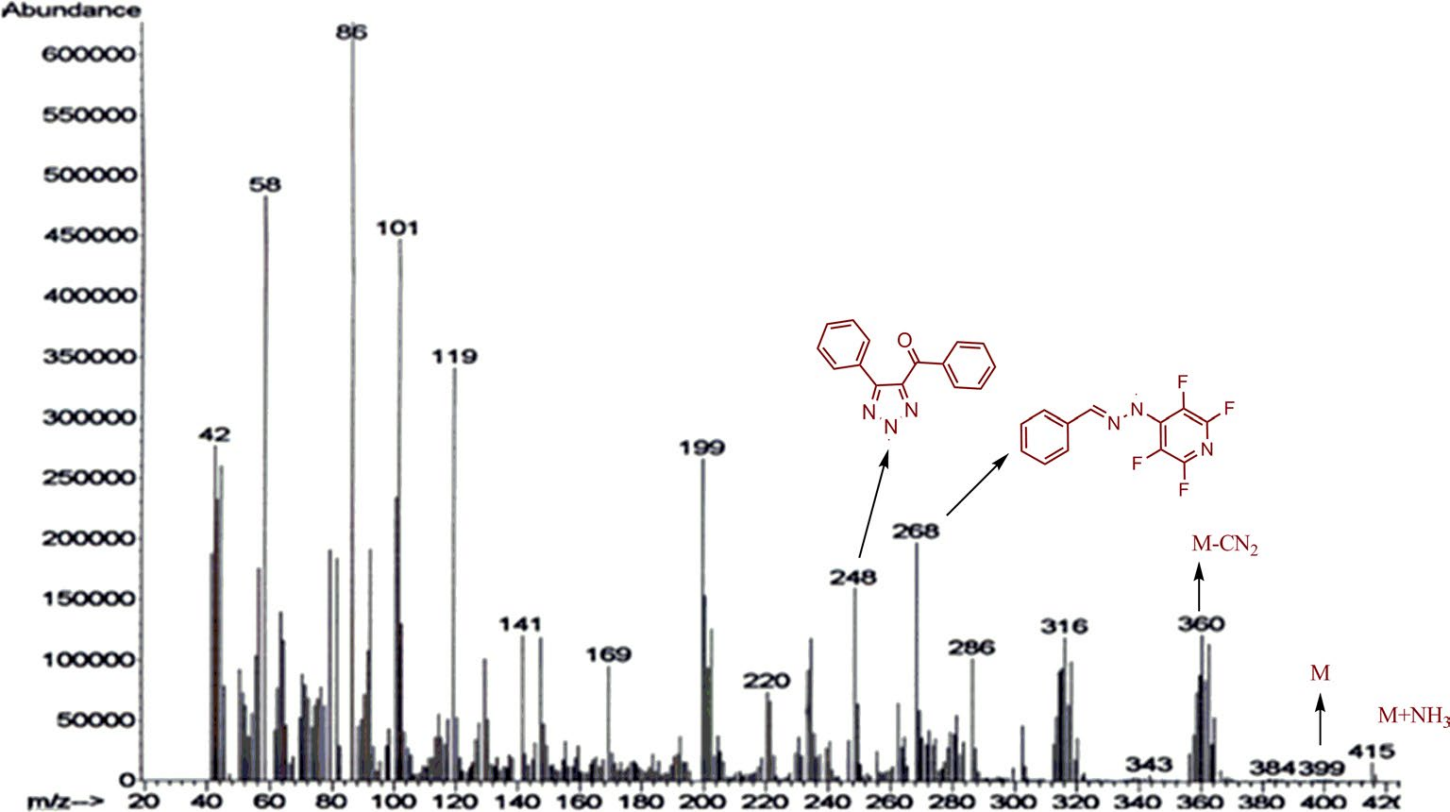
Mass spectra of **2a**

In ^19^F NMR analyze of **2a** exhibited two peaks for fluorine’s, a peak is observed at down field of doublet of doublet at δ = −89.54 (J = 24, 28 Hz) for F-2,6 (ortho positions) and also, a doublet of doublet is remarked at up field −144.83 (J = 20, 8 Hz) for F-3,5 (meta positions). A part of the ^19^F NMR spectrum of **2a** is shown in Fig. 3. ^19^F NMR analysis of **2a** confirmed that the nucleophilic substitution had occurred at the 4-position of pyridine ring. The mass spectrum of **2a** displayed molecular ion peak (M^+^) at *m*/*z* = 399, which is consistent with the proposed structure. Other ion peak are shown in mass spectra of **2a** (Fig. 4).

The structure of compounds **2b** was confirmed by NMR spectroscopic data. In particular, ^19^F-NMR spectroscopy show the chemical shift of fluorine atoms attached to the ortho and meta position are observed respectively at −95 and −153 ppm. The ^1^H NMR spectra of compound **2b** showed an H broad signal at 4.5 ppm for OCH_3_ group, and the protons of the phenyl ring were observed at δ = 7.6–8.6 ppm. The mass spectrum of **2b** displayed the molecular ion peak (M − 1) at *m*/*z* = 427, which is consistent with the proposed structure.

## Experimental

All reagents and solvents were purchased from Aldrich and Merck were used without further purification. The ^1^H and ^13^C NMR spectra were obtained on Bruker with DMSO as a solvent (^1^H NMR at 300 MHz and ^13^C NMR at 75 MHz). In the ^19^F-NMR spectra (282 MHz), up field shifts were quoted as negative and referenced to CFCl_3_. Mass spectra were taken by a Micro mass Platform II: EI mode (70 eV).

### General procedure for the preparation of 2-(tetrafluoropyridin-4-yl)-1,2,3-triazole

A mixture of chalcone (1 mmol), sodium azide (1 mmol) and CuO (2.5 mol%) were stirred in DMF (3 mL) for 20 h at 100 °C. After completion of the reaction as indicated by TLC pentafluoropyridine (1 mmol) was added to the mixture and the reaction continued at 100 °C for 5 h. Following, to the reaction mixture was added water 4 mL, and extracted with ethyl acetate and dichloromethane (3 × 5 mL). The solvent was removed in vacuo, and the crude product was purified by column chromatography using ethyl acetate/*n*-hexane (2/10) to give the pure product.

### (2-(Perfluoropyridin-4-yl)-5-phenyl-2*H*-1,2,3-triazol-4-yl)(phenyl)methanone (**2a**)

(0.24 g, 65 %) as brown solid; mp 240–245 °C decompose. ^1^H NMR (DMSO): δ (ppm) 7.96–8.41 (10H, m, Ar–H); ^19^F NMR (DMSO): δ (ppm) −89.5 (2F, m, J_FF_ 23, F-2,6), −144.8 (2F, m, J_FF_ 20, F-3,5); ^13^C NMR (DMSO): δ (ppm) 126.81, 126.96, 127.40, 128.14, 128.18, 128.24, 128.29, 128.66, 128.74, 128.86, 128.92, 129.23, 129.44, 129.52, 129.77, 129.86, 170.31 ppm. MS (EI), *m*/*z* (%) = 415 [M+NH_3_]^+^, 399 (M), 384, 360, 343, 316, 286, 268, 248, 220, 199, 169, 141, 119, 101, 86, 58, 42.

### (5-(4-Methoxyphenyl)-2-(perfluoropyridin-4-yl)-2*H*-1,2,3-triazol-4-yl)(phenyl)methanone (**2b**)

(0.20 g, 60 %) as a yellow solid; mp 265–270 °C decompose. ^1^H NMR (DMSO): δ (ppm) 4.5 (3H, OCH_3_) 7.60–8.61 (10H, m, Ar–H); ^19^F NMR (DMSO): δ (ppm) −95.0 (2F, m, F-2,6), −153.0 (2F, m, F-3,5); ^13^C NMR (DMSO): δ (ppm) 114.29, 115.54, 121.54, 122.94, 123.23, 127.82, 130.35, 130.85, 140.85, 141.22, 157.44 ppm. MS (EI), *m*/*z* (%) = 427 [M−1], 321, 282, 165, 91.

## Conclusion

Diels–Alder cycloaddition reaction of chalcone with azide in the presence of CuO as catalyst gives 4,5-disubstituted 1,2,3-triazoles, in reaction with pentafluoropyridine give 4,5-disubstituted-2 tetrafluoropyridyl-1,2,3-triazoles.

## References

[CR1] Van Ba N, Donald JB (2012). Preparation of p-substituted tetrafluoropyridyl derivatives via the tetrafluoropyridylcopper reagent. J Fluor Chem.

[CR2] Bruce ES (2001). Fluorine substituent effects (on bioactivity). J Fluor Chem.

[CR3] Hadjar B, Richard DC, Philip RH, Graham S (2001). Multi-substituted heterocycles. J Fluor Chem.

[CR4] Iwao O (2009). Fluorine in medicinal chemistry and chemical biology.

[CR5] Jingjing Wu, Dan F, Song C (2014). Synthesis of polyfluoroaryl-containing 1,2,3-triazoles by reaction of polyfluoroarenes, sodium azide and active methylene ketones/esters. J Fluor Chem.

[CR6] Kenneth LK (2006). Fluorine in medicinal chemistry: recent therapeutic applications of fluorinated small molecules. J Fluor Chem.

[CR7] Khalil B, Reza H, Malek Taher M (2015). Synthesis of 2,3,5,6-tetrafluoro-pyridine derivatives from reaction of pentafluoropyridine with malononitrile, piperazine and tetrazole-5-thiol. SpringerPlus.

[CR8] Khalil B, Reza H, Malek Taher M (2016). Reaction of hydroxylated naphtoquinones/antraquinones with pentafluoropyridine. SpringerPlus.

[CR9] Mark AF, Graham P, Graham S, Andrei SB (2013). ^19^F and ^13^C GIAO-NMR chemical shifts for the identification of perfluoro-quinoline and-isoquinoline derivatives. J Fluor Chem.

[CR10] Matthew WC, Emma LP, Graham P, Rachel S, Graham S, Ian W, Dmitrii SY, Judith AKH, John AC, David DM (2010). Annelation of perfluorinated heteroaromatic systems by 1,3-dicarbonyl derivatives. Tetrahedron.

[CR11] Reza RK, Graham S, Dmitrii SY, Judith AKH (2008). Macrocycles from pentafluoropyridine and tetrafluoropyrimidine. J Fluor Chem.

[CR12] Yuanqing Z, Xiaolong L, Jihui L, Jinying C, Xu M, Mingming Z, Baohua C (2012). CuO-promoted construction of N-2-aryl-substituted-1,2,3-triazoles via azide-chalcone oxidative cycloaddition and post-triazole arylation. Org Lett.

